# Clinical Practice Guideline for Best Practice Management of Pediatric Patients by Chiropractors: Results of a Delphi Consensus Process

**DOI:** 10.1089/jicm.2023.0010

**Published:** 2024-03-15

**Authors:** Genevieve Keating, Cheryl Hawk, Lyndon Amorin-Woods, Deisy Amorin-Woods, Sharon Vallone, Ronald Farabaugh, Angela Todd, Randy Ferrance, Jessie Young, Stephanie O'Neill Bhogal, Helen Sexton, Helen Alevaki, Joyce Miller, Gregory Parkin-Smith, Alec Schielke, Andrew Robinson, Robyn Thompson

**Affiliations:** ^1^ Fielding Graduate University, Santa Barbara, CA, USA.; ^2^ Private Practice, Melbourne, Australia.; ^3^ US-Clinical Compass, Lexington SC, USA.; ^4^ Texas Chiropractic College, TX, USA.; ^5^ School of Allied Health, Murdoch University, Murdoch, Australia.; ^6^ Private Practice, Perth, Australia.; ^7^ Edith Cowan University, Joondalup, Australia.; ^8^ Private Practice, Insight Counselling, Perth, Australia.; ^9^ Private Practice, Kidspace, Tolland, CT, USA.; ^10^ Advanced Medicine Integration Group, Columbus, OH, USA.; ^11^ Clinical Compass, Columbus, OH, USA.; ^12^ Private Practice, Sale, Australia.; ^13^ Aus-ACA, Sale, Australia.; ^14^ Private Practice, Richmond, VA, USA.; ^15^ US-ACA.; ^16^ Private Practice, Olympia, WA, USA.; ^17^ Life University, Marietta, GA, USA.; ^18^ Private Practice, Bendigo, Australia.; ^19^ Anglo European College of Chiropractic, Bournemouth, United Kingdom.; ^20^ School of Allied Health, Murdoch University, Murdoch, Australia.; ^21^ Palmer College of Chiropractic, Davenport, IA, USA.; ^22^ University of Tasmania, Hobart, Australia.; ^23^ Private Practice, The Thompson Method Breastfeeding, Melbourne, Australia.; ^24^ Australian Catholic University, Fitzroy, Australia.

**Keywords:** adolescent, chiropractic, child, infant, spine, spinal, manipulation, mobilization, pediatrics

## Abstract

**Objective:**

To build upon existing recommendations on best practices for chiropractic management of children by conducting a formal consensus process and best evidence synthesis.

**Design:**

Best practice guide based on recommendations from current best available evidence and formal consensus of a panel of experienced practitioners, consumers, and experts for chiropractic management of pediatric patients.

**Methods:**

Synthesis of results of a literature search to inform the development of recommendations from a multidisciplinary steering committee, including experts in pediatrics, followed by a formal Delphi panel consensus process.

**Results:**

The consensus process was conducted June to August 2022. All 60 panelists completed the process and reached at least 80% consensus on all recommendations after three Delphi rounds. Recommendations for best practices for chiropractic care for children addressed these aspects of the clinical encounter: patient communication, including informed consent; appropriate clinical history, including health habits; appropriate physical examination procedures; red flags/contraindications to chiropractic care and/or spinal manipulation; aspects of chiropractic management of pediatric patients, including infants; modifications of spinal manipulation and other manual procedures for pediatric patients; appropriate referral and comanagement; and appropriate health promotion and disease prevention practices.

**Conclusion:**

This set of recommendations represents a general framework for an evidence-informed and reasonable approach to the management of pediatric patients by chiropractors.

## Introduction

Chiropractors are concerned with the assessment, diagnosis, treatment, and prevention of disorders of the neuromusculoskeletal system and the potential effects of these disorders on general health for people of all ages.^1,2^ Although other types of health care providers, such as physical therapists, treat musculoskeletal symptoms in children (patients <age 18),^3^ chiropractic is the most common complementary and integrative medicine practice used by children in the United States^4^ and is also commonly used by children around the world, including in Australia.^5^ In Australia, the best estimates are that chiropractors provide care to more than 30,000 child patients (0–18 years old) every week.^6^ According to a 2017 scoping review, ∼8% of chiropractic patients are 18 years of age and under.^7^ Manual care for children is most often sought for the treatment of musculoskeletal (MSK) conditions.^8^

In 2019, in Victoria, Australia, a state government inquiry into chiropractic care of children under 12 was conducted by Safer Care Victoria (SCV).^9^ SCV sought input from consumers and received 22,043 responses from parents who had accessed chiropractic care for their child(ren) under 12 years of age. While most respondents sought chiropractic care for musculoskeletal conditions, up to 40% were related to developmental concerns.^10^ Most of the children who presented to chiropractors were also under the care of other health professionals for their presenting concerns. The other health professionals consulted were primarily general (medical) practitioners, maternal and child health nurses, and medical specialists. Over 99% of parents of children consulting chiropractors in the report felt well informed and involved in the decisions about the care. According to the parents, 98.4% said they had noticed, or their child had reported, an improvement after the care was provided.^11^

Another major part of the SVC inquiry was the commission of a Cochrane Collaboration review of the efficacy and safety of chiropractic spinal manipulation for children.^12^ This Cochrane Review was not published in a scientific journal but played a key role in determining the SCV recommendations.^13^ The review, in common with other reviews, noted that adverse events (AEs) are very rare in children receiving chiropractic manipulation.^8,12,14^ A 2023 retrospective analysis of 54,846 patients, involving 960,140 chiropractic treatment visits, found that none of the 39 AEs reported occurred in children; the median patient age of those with AEs being 50.8 years.^15^

However, because of the lack of definitive evidence of efficacy and effectiveness for various conditions, the Cochrane review concluded that spinal manipulation is not recommended for children under 12, for a number of conditions, or for general wellness.^12,13^ It is important to note that the Cochrane review defined “spinal manipulation” as “any technique delivered by any health professional that involves a high-velocity, low-amplitude (HVLA) thrust beyond the physiological range of motion, impacting the spine, within the limits of anatomical integrity.”^12(p.4)^ The SCV report refers to a statement by the authors of one of the primary source documents cited by the Cochrane Collaboration that chiropractors who treat children modify both biomechanical force and speed to accommodate the child's age and physical development so they are often not using an HVLA thrust.^16^ Quite apart from the recommendations of SCV, chiropractors have also acknowledged the pressing need for research on chiropractic care for children.^17,18^

Chiropractic, in common with other manual care professions, is responsible for ethical and safe practice, which requires cultivating and mastering both an academic foundation and clinical expertise.^1^ Chiropractic undergraduate education involves the study of the unique anatomy and physiology of the child as well as the modification of evaluative and therapeutic procedures as applied to this unique population when addressing neuromusculoskeletal problems and the potential effect on overall health and wellbeing. In Australia, chiropractic education specifically requires graduates to adapt practice according to varying patient needs across the human lifespan, including the need for care and management options to be tailored for individuals.^19^^(p.13)^

By nature of training and scope, chiropractors also address public health issues and supportive measures for healthy human development during the clinical encounter.^20^ These may include advice regarding injury prevention, healthy diet, physical activity needs, sleep advice, reduced screen time (including mobile/cell phone use), healthy social relationships, and vigilance around childhood trauma.^21–23^ It has been found that, in general, children with a decreased health-related quality of life have higher utilization of complementary and integrative medicine.^24,25^

In 2009 and 2016, Hawk et al. performed consensus processes, gathering expert opinion on best practices for the chiropractic care of children.^21,22^ The resulting documents have helped provide practitioners with guidelines. They have also been helpful to other providers, the public, and third-party payers in demonstrating that the chiropractic profession has high standards for the care of children. However, the most recent of these documents were based on the literature published before 2015, so in keeping with recommendations for guidelines^26,27^ and to address a key recommendation of the SCV regarding the safety of chiropractic care, we conducted this project.

## Aims and Purpose

This project aimed to build upon the existing recommendations on best practices for the chiropractic management of children by conducting a formal Delphi consensus process and best evidence synthesis. Our new emphases were on providing the safest possible chiropractic care for children and providing appropriate health promotion and disease prevention practices. The process was based on the 2016 best practices^22^ recommendations and the findings of the SCV Cochrane review with a brief update of the SCV's review of the safety of spinal manipulation for children.

## Methods

### Human subject considerations

No data were collected from any patients or parents of patients. The only personal data collected were from the Delphi consensus panel experts. The project was approved by the Institutional Review Board of the Project Director's (C.H.) institution before collection of any information from these individuals. As part of this process, the panelists signed a consent form confirming their voluntary and uncompensated participation. They also signed a permission-to-acknowledge form before submission of the article for publication.

### Steering committee

The steering committee (SC) was composed of experienced clinicians and academics from health professions involved in the care of children, and several also represented chiropractic organization stakeholders.

The SC's role in the project was to provide input on evidence, contribute to drafting the seed recommendations, revise the recommendations based on the Delphi panel's comments and ratings to achieve consensus, and contribute to the development of the final article.

Table 1 summarizes the expertise and affiliations of the SC. Of the 16 members, 13 are chiropractors; of those, 2 are also cross-trained as medical physicians (1 Medical Pediatrician); other professions in which the chiropractors are cross-trained are health education, lactation consulting, massage therapy, and public health.

**Table 1.** Composition of the Steering Committee

Expertise	Profession	Organization
Project Director: Research methodology (PhD), health education, chiropractic education	Chiropractor Massage Therapist	U.S. Clinical Compass; U.S. Texas Chiropractic College
SC Chair: Early childhood development (PhD), chiropractic education, research, chiropractic clinical practice	Chiropractor	Aus-ACA
SC members		
Clinical practice	Chiropractor	Chair, Pediatric group of Australian Institute of Chiropractic Education
Clinical practice, public health, chiropractic education, research	Chiropractor	Murdoch University, Perth Australia
Clinical practice	Chiropractor	U.S. Clinical Compass
Clinical practice, research, Musculoskeletal Health of Infant (PhD)	Chiropractor	
Clinical practice, chiropractic education, pediatrics	Chiropractor	ICA Pediatrics Council; Life University
Clinical practice	Chiropractor	
Clinical practice, pediatrics, research	Chiropractor	Director, National Board of Aus-ACA
Clinical practice, journal editor, pediatrics, research	Chiropractor	ICA Pediatrics Council Editor, Journal of Clinical Chiropractic Pediatrics
Clinical practice, lactation consultant	Chiropractor, Lactation Consultant	U.S.-ACA Pediatrics Council
Clinical practice, research	Chiropractor, Medical Physician	
Clinical practice, pediatric medicine, internal medicine	Chiropractor, Medical Pediatrician and Internist	
Clinical practice midwifery, Breastfeeding consultant PhD	Midwife, Breastfeeding Consultant	
Clinical practice PhD	Registered Nurse	Consumer representative
Clinical counseling practice, research, education	Family Therapist, Mental Health Practitioner, Lecturer	Consumer representative, Edith Cowan University, Associate Editor Aust & New Zealand J Family Ther

ICA, International Chiropractors Association; SC, steering committee; U.S.-ACA, American Chiropractic Association.

Those SC members who are not chiropractors represent family therapy, psychology, social work (1), midwifery/lactation consulting (1), and nursing (1). Five of the SC members have PhDs; topics for these PhDs are Early Childhood Development; Musculoskeletal Health of the Infant; Preventive Medicine; Midwifery, Maternal, and Child Health; and Nursing. Four are faculty at chiropractic institutions.

Eight members have advanced training/certification in pediatrics. For purposes of stakeholder representation, these chiropractic organizations are represented by SC members: Australian Chiropractors Association (Aus-ACA), American Chiropractic Association (U.S.-ACA), U.S. Clinical Compass, and International Chiropractors Association (ICA). Two nonchiropractic SC members represented the consumer perspective.

### Approach to the literature search

We began the project with two seed documents. First was the 2016 set of recommendations on best practices for chiropractic care for children, which had been itself an update of recommendations published in 2009.^21,22^ We used this set of recommendations as a starting point for this project and included updates and expansion based on current evidence and issues.

The second document was the 2019 systematic review prepared by Cochrane Australia for SCV.^12^ Although this rapid review of the effectiveness, efficacy, and safety of spinal manipulation for children was not published in a peer-reviewed journal, as an official government publication, we used its findings as a primary resource for developing our recommendations. The Cochrane Australia Review concluded that, although AEs were rare, they could not draw conclusions about the safety and effectiveness of spinal manipulation for children under 12 because of the paucity of studies and lack of specific treatment information^12^

For this reason, we decided that until more definitive research is available, it is still premature to make recommendations on the treatment of specific conditions in children. Therefore, because studies on the effectiveness and efficacy of even the most common conditions for which children seek chiropractic care are likely to remain sparse in the immediate future, we made the decision to focus our evidence-based recommendations on ensuring that chiropractic care is delivered as safely as possible.

### Primary search strategy

We conducted a literature search to update the evidence on safety obtained from the Cochrane Australia Review, which ended in June 2019. Our research question was: “What are the adverse events associated with chiropractic care, including spinal manipulation among children (<18 years of age)?”^22^ To not be redundant with the results of the Cochrane Australia Review, we used a start date after that study's search ended. Since similar searches we have used in the past have found that high-quality articles were not missed in PubMed, we did not include other databases in the search. In February 2022, we searched PubMed from July 1, 2019 to January 31, 2022 using these terms: (“Manipulation, Spinal” OR “Manipulation, Chiropractic” OR Chiropractic OR “manual therapy” OR “manual therapies”) AND (pediatric OR pediatrics OR child OR infant OR adolescent OR newborn) AND (safety OR “adverse effects” OR “adverse events”)

### Eligibility criteria for articles on AEs and safety

Inclusion criteria were:

- Published July 1, 2019 to January 31, 2022
- Human subjects
- English language
- Chiropractic spinal manipulation was the treatment/intervention
- Study population was children (<18 years)
- Included information on AEs/safety

Exclusion criteria were:

- Commentaries/editorials/letters
- Nonpeer-reviewed publications
- Study protocols with no results
- Surveys and other cross-sectional studies
- Conference abstracts

At least two investigators screened the search results for eligibility.

### Secondary (targeted) searches

As we developed the seed statements, which addressed each component of the clinical encounter, we conducted targeted searches for documentation. These were not formal and exhaustive searchers; we asked topic experts in our SC to also consult their personal libraries, using the same eligibility criteria as the previous search. We added an expanded section on health promotion and disease prevention practices recommended for all health professionals to this set of recommendations; thus, we searched recognized organizations, including the U.S. Preventive Services Task Force (USPSTF) and the U.S. Centers for Disease Control and Prevention (CDC), as well as a recently published clinical practice guideline (CPG) developed specifically for the role of chiropractic in health promotion and disease prevention for adults with musculoskeletal pain.^20,28,29^

### Evaluation of the quality of the evidence

In keeping with the precedent used by the Cochrane Australia Review, we did not assess the quality of the studies from the search for AEs related to spinal manipulative therapy (SMT). For evidence supporting recommendations on “best practices” for chiropractors treating children (which were identified through targeted searches and SC recommendations), we evaluated the quality of CPGs, systematic reviews/meta-analyses, randomized controlled trials (RCTs), and narrative reviews. Other types of articles were categorized as “lower level” quality of evidence and were not formally rated. Although rapid reviews are an increasingly popular method of assessing the literature,^30^ there is not yet agreement on the best set of quality assessment considerations, so we did not formally assess the quality of rapid reviews.

We evaluated CPGs using the AGREE-GRS (Global Rating Scale) (Table 2).^31^ We evaluated systematic reviews (Table 3) and RCTs (Table 4) using modified SIGN (Scottish Intercollegiate Guideline Network) checklists.^32^ We evaluated narrative reviews (Table 5) using SANRA (scale for the quality assessment of narrative review articles).^33^

**Table 2.** AGREE Global Rating Scale

Seven items using ordinal rating scale of 1 (lowest) to 7 (highest) quality; maximum sum of 49. Average score = total score/7. Quality interpreted as follows: High quality: average 6–7; acceptable quality: average 4–5; unacceptable quality: <4
1. Development process—rate overall quality of guideline development methods.
Appropriate stakeholders involved in the development of the guideline?
Evidentiary base developed systematically?
Recommendations consistent with the literature
2. Presentation style—rate overall quality of the guideline presentation.
Guideline well organized?
Recommendations easy to find?
3. Completeness of reporting—rate completeness of reporting.
Development process transparent and reproducible?
Was information to inform decision making complete?
4. Clinical validity—rate overall quality of the guideline recommendations.
Recommendations clinically sound?
Recommendations appropriate for the intended patients?
Overall assessment
1. Rate the overall quality of this guideline.
2. I would recommend this guideline for use in practice.
3. I would make use of a guideline of this quality in my professional decisions.

Aus-ACA, Australian Chiropractors Association.

**Table 3.** Systematic Review/Meta-Analysis (Modified Scottish Intercollegiate Guideline Network—Checklist)

	Item	Yes/no^a^
1	Research question clearly defined, and eligibility criteria listed.	
2	Comprehensive literature search conducted.	
3	At least two people selected studies.	
4	At least two people extracted data.	
5	Status of publication not used as an inclusion criterion.	
6	Excluded studies listed.	
7	Relevant characteristics of included studies provided.	
8	Quality of included studies assessed and reported.	
9	At least two people assessed quality of included studies.	
10	Appropriate methods used to combine individual study results.	
11	Likelihood of publication bias assessed appropriately.	
12	Conflicts of interest declared.	
	Total score^b^	

^a^ Rating: “Yes” = 1; “No” or unable to tell from the article = 0.

^b^ Scoring—sum of items as follows: 10–12 = high quality, low risk of bias; 6–9 = acceptable quality, moderate risk of bias; <6 = low quality, high risk of bias.

**Table 4.** Randomized Controlled Trial (Modified SIGN Checklist)

	Item	Yes/no^a^
1	Addressed an appropriate and clearly focused question.	
2	Group assignment randomized.	
3	Sample size justified by a power calculation.	
4	Investigators blinded to patients' group assignment.	
5	Patients blinded to group assignment.	
6	Groups were similar at the start of the trial.	
7	Only difference between groups was treatment of interest.	
8	Outcomes measured in a standard, valid, and reliable way.	
9	Power calculation was used and required sample size attained.	
10	Intention-to-treat analysis performed.	
	Total score^b^	

^a^ Rating: “Yes” = 1; “No” or unable to tell from the article = 0.

^b^ Items scored as follows: 9–10 = high quality, low risk of bias; 6–8 = acceptable quality, moderate risk of bias; <6 = low quality, high risk of bias.

**Table 5.** Scale for the Assessment of Narrative Review Articles

Follow instructions for rating: https://researchintegrityjournal.biomedcentral.com/articles/10.1186/s41073-019-0064-8		
	Item	Rating: 0, 1 or 2^a^
1	Justification of the article's importance for the readership	
2	Statement of concrete aims or formulation of questions	
3	Description of the literature search	
4	Referencing	
5	Scientific reasoning	
6	Appropriate presentation of data	
	Total score^a^	

Notes on ratings—list by item number.

^a^ Quality rating as follows: 10–12 = High; 6–9 = Acceptable; <6 = Unacceptable.

At least two investigators (C.H., L.A.-W., D.A.-W., A.S.) rated each article; differences in ratings were resolved by discussion.

### Seed statement development

The SC used the 2016 best practices for chiropractic care for children document as a starting point to develop the seed statements.^22^ For this project, we expanded the brief section in the 2016 article on health promotion and disease prevention. We used the literature search results to inform the process of developing draft seed statements. Using an iterative process, the SC continued drafting the seed statements to achieve clarity and incorporate the best evidence to support them. When they reached agreement, the statements were then ready for the Delphi process.

### Delphi panel

All members of the SC were consulted to nominate Delphi panelists, taking into consideration a number of factors: (1) balancing experienced Delphi panelists with new panelists; (2) representing chiropractic and other professionals involved in health care for children; (3) representing both practitioners who specialize in chiropractic pediatrics and “generalist” practitioners; (4) including experts in research and academics as well as practitioners. Panelists were based on their practice characteristics (provided in a form accompanying their nomination as a panelist) and their Curriculum Vitae; they were invited to participate after approval by the SC.

### Rating of seed statements in modified Delphi process

We used the RAND/University of California at Los Angeles (RAND-UCLA) methodology for modified Delphi processes for rating the appropriateness of the described procedure.^34^ It “generally involves multiple rounds, in which a questionnaire is sent to a group of experts who answer the questions anonymously. The results of the survey are then tabulated and reported back to the group, and each person is asked to answer the questionnaire again. This iterative process continues until there is a convergence of opinion on the subject or no further substantial changes in the replies are elicited.”^34(p. 6)^

As per the standard methodology, panelists rated each seed statement using an ordinal scale of 1–9 (highly inappropriate to highly appropriate).^34^ Instructions were to rate the statements as^34^:


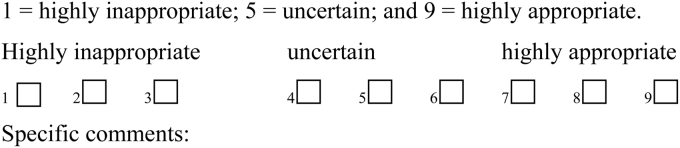


“Appropriateness” is defined by the RAND-UCLA methodology as meaning that the patient's expected health benefit is greater than any expected negative consequences by a sufficiently wide margin that it is worth doing, without considering cost.^34^ If panelists intended to rate a statement as inappropriate (rating 1–3), we required them to provide a reason and, if possible, cite peer-reviewed articles in support. We considered this necessary for the SC to make an evidence-informed revision of the statement and accurately represent the panel's input. If the panelist did not supply a reason, we considered the response a missing value. Delphi panelists were provided with all supporting citations; upon request, they were provided with the full-text documents.

### Modified Delphi rounds, rating system, and data analysis

The project coordinator entered all numerical response data into an SPSS file for analysis (median and percent agreement). She entered text data (comments) into a Word table, organized by panelist identification number (ID), statement ID, and rating. All data, both numeric and text, were identified only by a code number when circulated to the panelists and the SC. The project director collapsed the 1–9 scale into three categories, “inappropriate” (1–3), “undecided” (4–6), and “appropriate (7–9), and analyzed this frequency data for the median rating and percent agreement. Statements not reaching 80% agreement for appropriateness (i.e., ratings 7–9) were revised by the SC, based upon the panelists' comments, and were recirculated until the panel reached at least 80% agreement.

## Results

### Literature search

#### Safety literature search

**FIG. 1.**
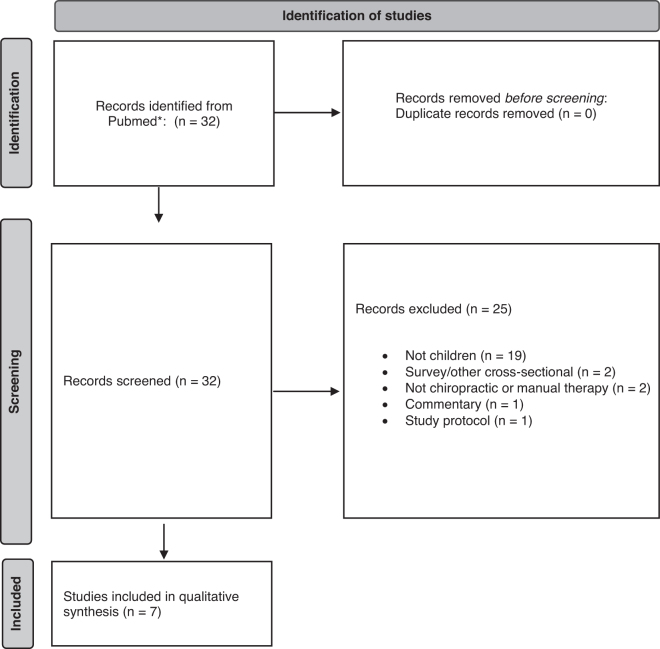
PRISMA flow diagram for safety and AEs literature search. *Excluded studies are listed in Supplementary materials. AEs, adverse events; PRISMA, Preferred Reporting Items for Systematic Reviews and Meta-Analyses.

The PubMed safety search (covering the period July 1st 2019–January 31st 2022) yielded 32 studies, with 25 excluded, as detailed in Figure 1 (excluded studies are listed in Supplementary materials) The remaining seven studies are detailed in Table 6.^8,35–40^

**Table 6.** Studies in Updated Safety Search

First author and year	Topic	Interventions	Design	Relevant findings
Ellwood 2020^36^	Conservative interventions for positional plagiocephaly and congenital muscular torticollis	MT, repositioning, helmet, orthotics	SR	All AEs reported were already reported in Cochrane Australia review.
Elwood 2020^35^	Common interventions for infantile colic	MT, probiotics, simethicone, PPI	SR	All AEs reported were already reported in Cochrane Australia review.
Paknejad 2019^38^	Traditional, complementary and alternative medicine in children with constipation	Herbal and traditional medicines, massage, reflexology, acupuncture, MT, kineseotaping	SR	Of 10 manual therapy studies, 2 reported on AEs and both of those reported no AEs.
Corso 2020^8^	The safety of SMT in children under 10 years	SMT, acupressure, MUA, proEFA supplement	RR	Most AEs mild Incidence 0.3%–22% True risk of SMT unknown
Pohlman 2020^39^	Comparison of active v. passive AE surveillance in pediatric ambulatory chiropractic care	Usual and customary chiropractic care (unspecified)	RCT	Estimated parent/DC-reported AEs 2% in active surveillance None required urgent medical care All were associated with irritability/crying, pain, fatigue, or self-care limitation
Vos 2021^40^	AEs associated with pediatric complementary and alternative medicine in the Netherlands: a national surveillance study	Bioresonance, cervical SMT, herbal therapy, homeopathy, kinesiology, nutritional supplements, energy medicine, massage	CO	32 AEs reported through active surveillance by 1300 Dutch pediatricians over 3 years serving >500,000 children/year One indirect AE related to chiropractic care due to delay of medical treatment; SMT was not involved.
Ghanim 2020^37^	Recurrent stroke in child with atlantoaxial instability^a^	Child had sustained a fall and received chiropractic SMT 1 year before onset of signs/symptoms	CR	No association or causal relationship indicated between SMT and recurrent strokes

^a^ Despite the title of this article, there was no evidence presented that it reported an AE directly related to chiropractic SMT.

AE, adverse events; CO, cohort study; CR, case report; MT, manual therapy; MUA, manipulation under anesthesia; PPI, protein pump inhibitor; RCT, randomized controlled trial; RR, rapid review; SMT, spinal manipulative therapy; SR, systematic review.

### Quality assessment

Table 7 lists the CPGs, systematic reviews, and narrative reviews rated for quality. Other study designs were considered lower-level evidence and were not rated. Seven of the eight guidelines were rated as high quality^41–47^; one was not rated because two of the investigators on this project were authors, and it was used as a seed document for information about health promotion and disease prevention.^20^ Of the four systematic reviews, three were rated as high quality ^48–50^ and one as acceptable.^12^ Of the four narrative reviews, three were rated as high quality ^16,48,51^ and one as acceptable.^52^

**Table 7.** Quality Assessment of Clinical Practice Guidelines, Systematic Reviews and Narrative Reviews

Topic	Title	First author	Year	Quality*^a^*
CPGs				
Adolescent idiopathic scoliosis	2016 SOSORT guidelines: orthopedic and rehabilitation treatment of idiopathic scoliosis during growth	Negrini^43^	2016	H
Adolescent idiopathic scoliosis	Screening for adolescent idiopathic scoliosis: USPSTF recommendation statement.	Grossman^41^	2018	H^b^
Child abuse	Preventing Child Maltreatment: a Guide to Taking Action and Generating Evidence.	WHO^47^	2006	H^b^
Chiropractic care and health promotion	The role of chiropractic care in providing health promotion and clinical preventive services for adult patients with musculoskeletal pain: a CPG	Hawk^20^	2021	(Not rated)^c^
Obesity	Screening for obesity in children and adolescents: USPSTF recommendation Statement.	USPSTF^46^	2017	H^b^
Skin cancer prevention	Behavioral counseling to prevent skin cancer: USPSTF recommendation statement.	USPSTF^106^	2018	H^b^
Spine care pathway	Global spine care initiative: care pathway for people with spine-related concerns.	Haldeman^42^	2018	H
Tobacco cessation	Recommendations from the USPSTF for prevention and cessation of tobacco use in children and adolescents.	USPSTF^107^	2020	H^b^
Systematic reviews				
Spinal manipulation	Systematic review of spinal manipulation in children.	Green^12^	2019	A
Spinal manipulation	SMT in infants, children and adolescents: systematic review and meta-analysis on treatment indication, technique, and outcomes	Driehuis^48^	2019	H
Manual therapy	Manual therapy for the pediatric population: a systematic review	Parnell Prevost^49^	2019	H
Adolescent idiopathic scoliosis	SMT for adolescent idiopathic scoliosis: a systematic review	Théroux^50^	2017	H
Narrative reviews				
Child abuse	A review of the literature on good practice considerations for initial health system response to child and adolescent sexual abuse.	Broaddus-Shea^92^	2021	H
Diagnostic imaging	Current evidence for spinal X-ray use in the chiropractic profession: a narrative review.	Jenkins^52^	2018	A
Safety	AEs due to chiropractic and other manual therapies for infants and children: a review of the literature	Todd^51^	2014	H
Safety	Forces of commonly used chiropractic techniques for children: a review of the literature	Todd^16^	2016	H

^a^ Quality rating: H = High; A = Acceptable.

^b^ Based on the published methodologies for USPSTF and WHO, we classified their guidelines as high quality.

^c^ We did not rate this guideline because two of its principal authors are investigators on this project.

CPGs, clinical practice guidelines; WHO, World Health Organization; USPSTF, United States Preventive Services Task Force.

### Delphi panel characteristics

Of the 60 panelists, 65% (39) were female, and 35% (21) were male. All but two were of white/Caucasian ethnicity; one reported as Asian/Pacific Islander, and one multi/biracial. All 60 were chiropractors; 1 was also a physiotherapist, and 1 was also a massage therapist. Five had additional Master's degrees, and 4 also had PhDs. The mean years practicing in their profession was 22 (range, 1–53 years). In terms of geography, 55% (33) were from Australia (6 of 6 states: NSW, QLD, SA, TAS, WA, VIC); 28% (17) were from the U.S. (13 of 50 states: AZ, CA, FL, IA, IL, MO, MS, MN, NY, OK, OR, TX, WA); and 17% (10) were from countries other than Australia and the U.S.: Denmark, England, Ireland, Malaysia, New Zealand, and South Africa. Nine panelists were faculty at a chiropractic institution, 3 at a nonchiropractic institution, and four at a chiropractic program within a nonchiropractic institution. Fifty-five percent (33) had some type of postgraduate certification in pediatrics.

For the 53 panelists who are practicing clinicians, their mean years in practice was 22 (range, 1–43 years); the mean number of estimated pre-COVID (coronavirus disease 2019) patients per week was 89 (range, 0–280); and mean estimated percent of those patients who were under age 18 was 39% (range, 0%–100%).

### Delphi process

The Delphi process was conducted June to August 2022. In the first round, 66 of the 70 seed statements reached at least 80% consensus. In the second round, 2 of the 4 revised statements from Round 1 reached 80% consensus. In the third round, the last two statements reached consensus. All 60 panelists participated in each of the 3 rounds, although 3 declined to be acknowledged.

### Seed statements

The following are the recommendations for best practices for chiropractic care for children.

#### Introduction

In1. The purpose of these recommendations is to ensure the health and safety of chiropractic patients under 18 years of age by identifying the components of the most appropriate clinical approach to chiropractic care in the pediatric age group.

In2. Weigh the potential benefits of an intervention against potential health risks and financial and/or time costs.^53–55^

In3. Consider the anatomical, physiological, developmental, social, and psychological differences between children and adults to determine the application and appropriateness of health care evaluation and interventions.^22,56,57^

#### Patient communication

CO1. Gather relevant clinical information during the case history of a child using a flexible approach to establish rapport while collecting information.^22,56,58^

CO2. Communicate in an age- and developmentally appropriate manner to help a child actively engage in the clinical encounter^56,58^ as much as possible, incorporating relevant strategies and use of resources (using words, signs, or toys) to help a child feel at ease.^4,59–61^

CO3. During treatment, be sensitive to the child's cues when they communicate feelings (examples: fear, pleasure) or sensations (examples: ticklish, pain).^59,60^

CO4. In pediatric practice it is important to establish trust and rapport with both parents/caregivers and the child during the clinical encounter in a safe, inclusive, and nonjudgmental environment.^62^ This requires not only sensitivity to the language being used but also the nonverbal elements of the exchange.^56,59,60^

CO5. Be sensitive to cultural, religious, and gender considerations in patient dialog to optimize reaching an appropriate diagnosis, designing an effective and safe treatment plan and gaining consent for the mutually agreed plan.^63^

#### Informed consent

IC1. Ensure that an informed consent process is completed with the parent/guardian and the child if age- and developmentally appropriate before initiating any assessment, examination, or treatment. Obtain a signed informed consent form from the parent or legal guardian and document it in the patient's record. The informed consent process consists of explaining the recommended care, including benefits, potential risks, and alternative treatment options.^56^

IC2. Explain procedures in clear and simple terms; answer both the parent/guardian's and child's questions, to ensure that their consent is fully informed and voluntary.^56^

IC3. Obtain verbal consent from the child, if developmentally appropriate.^61^

IC4. Explain the diagnosis and prognosis to the parent/guardian and child in age-appropriate, understandable wording.^56^

IC5. Explain the proposed management/treatment plan and any possible risks, benefits, financial costs, and alternative treatment options (including natural history).^51^ When suggesting treatments for which current evidence is not definitive, refer to the “Chiropractic Management of Pediatric Patients” section below for specific language to explain such to parents/guardian.^8,56^

IC6. Consent should be freely given without coercion or pressure.^64^

IC7. Explain that the patient, parent, or guardian may withdraw consent at any time.

IC8. Provide the patient/parents with opportunities to ask questions at all stages of the informed consent, discussion of treatment options, assessment/examination, and treatment.

#### Clinical history

HE1^*^. In a comprehensive case history at the initial visit, include a review of systems, developmental milestones, family history, health care history, concurrent health care and medication use.^56,58^ Specific items include^65^:

- Onset of pain/symptoms
- Mechanism of injury or trauma
- Symptom parameters (timing, location, quality, frequency, intensity, duration, and radiation)
- Family history of relevant conditions
- Social/behavioral health history (such as activities, social/emotional stress, conflicts or abuse; depression, including suicidal ideation; gender identity)^63^
- Intellectual and/or behavioral difficulties, including presence of learning difficulties, attention-deficit/hyperactivity disorder (ADHD/ADD) and autism spectrum traits. Determine the relevance and effect of these difficulties on consent, history taking, delivery of treatment, and the type/style of treatment
- Growth, physical development, and milestones
- Health care history, including current and previous treatment for presenting complaint

HE2^*^. Elicit information on general health habits, including breastfeeding, diet, and dietary issues such as eating disorders,^66^ sleep, physical activity, and injuries.

HE3^*^. Review relevant prenatal events, including the health of the mother, as well as a review of the birth history (e.g., gestational age, birthweight, perinatal complications).^56^

HE4^*^. Obtain case history information from the child, if possible, to assist in developing an appropriate management plan.^61^

#### Physical examination

PE1. Use clinically relevant and valid examination procedures to move from a working diagnosis, which is based on the history, to a short list of differential diagnoses.^22,56,58,65^

PE2. Assess vital signs pertinent to their health and chief complaints in an age-appropriate manner as part of the initial examination and at reassessment intervals.^56,58^

PE3. Be familiar with the World Health Organization (WHO) growth charts (for children up to age 2) and CDC growth charts (children 2 years and older) and use when clinically applicable to monitor growth.

PE4. Refer any necessary diagnostic or examination procedures outside the chiropractors' clinical skills, scope of practice, or experience to an appropriately qualified health professional with whom to coordinate care.^1^

PE5. The neuromusculoskeletal examination should include^65^:

- Inspection and palpation for lesions, pain source, or masses
- Developmentally appropriate assessment of movement and gait
- Range of motion assessment of the spine and extremities, as relevant
- Assessment of joint motion as relevant
- Orthopedic and neurologic evaluation
- Postural assessment
- Assessment of organ systems when indicated such as eyes, ears, nose, throat, heart, lungs, and abdomen.

PE6. Conduct an age-appropriate developmental assessment, which, depending on clinical relevance, may include: balance and gait, fundamental developmental milestones, cranial nerve examination,^22,56,58,65,67^ muscle tone, primitive reflexes, postural reflexes, and gross and fine motor development.^68^

PE7. Refer children with possible developmental delay for further assessment as necessary.^69^

#### Red flags in pediatric patients

RF1. If the history and/or examination reveal “red flags” indicating serious conditions, refer the child to an appropriate provider for further diagnosis and/or care (Table 8).^22,56,58,65^

**Table 8.** Signs and/or Symptoms Suggestive of Emergent Condition for Which Immediate Medical Referral Is Indicated

Infants and very young children only:
Acute weight loss exceeding 5% of body weight
Bulging or sunken fontanelle
Fever >38°C (100.4°F) in a child <90 days of age
Inability to rouse the child
Persistent inconsolable high-pitched crying or a weak cry with drowsiness
Respiratory distress in neonates
Signs of dehydration and/or decreased fluid intake of 50% or greater over a period of 24 h
Children of any age:
Altered mental status, signs of dehydration, abdominal pain, or “fruity breath” in a child with diabetes
Bile-stained vomiting
Bone fracture or dislocation
Cold, pale distal lower extremities and or oral cyanosis
Convulsions, particularly if no prior history or associated with head trauma
Dyspnea, which may be accompanied by nasal flaring or significant increase in respiratory rate
Fecal blood
Fever of 40 degrees centigrade (104°F) or higher, particularly if spiking
Fever, chest pain, altered mental status, or other neurological findings in a child with Sickle Cell Disease
Hot, swollen, tender joints, especially if the child refuses to bear weight
Other orthopedic emergencies such as slipped femoral epiphysis or Perthes' Disease
Pallor
Persistent vomiting
Petechial or purpuric rash
Sudden onset or persisting acute abdominal symptoms
Signs/symptoms suggestive of potentially serious illness for which appropriate referral and/or comanagement are indicated:
Loss of developmental milestones
New onset strabismus
Parent suspects substance abuse
Persistent diarrhea
Persistent vomiting
Personality change
Positive neurological signs such as Babinski, Hoffman's, absent reflexes, motor weakness
Recurrent fevers
Scoliosis greater than 20°
Slurred speech
Suicidal ideation
Suspicion that child or primary care giver could be a victim of domestic violence
Unexplained bruising without trauma or suspicion of child abuse
Unexplained weight loss

#### Diagnostic imaging

DI1. Consider referral for radiographic examination in the presence of a history of trauma, suspicion of serious pathology, and/or positive results of screening assessment of scoliosis.^52,65^

DI2. In the absence of red flags, do not use routine or repeat radiographs for the evaluation of the structure and function of the spine.^52,70^

DI3. Where there are indicators of pathology or conditions that warrant further investigation, appropriate referral for assessment should be made in a timely manner.^65^

#### Chiropractic management of pediatric patients (See specific recommendations for infants below)

C1. There are four basic chiropractic management approaches to the care of a child: (1) sole management by a chiropractor, (2) independent concurrent care by a chiropractor and other provider(s), (3) comanagement with other appropriate health care providers, and (4) referral to another registered/licensed or certified health care provider/specialist.^1,22,42^

C2. Follow the principles of evidence-based practice, which are to make clinical judgments based on the best available evidence combined with clinical experience and the patient's preferences.^22,56,71,72^

C3. Children may present to chiropractic practices with various conditions and developmental concerns not directly related to the neuromusculoskeletal system. There is a paucity of high-level research evidence for the effectiveness of spinal manipulation and/or chiropractic care for such conditions.^8,12,48,49,73^ However, the absence of research evidence does not equate to evidence of absence and subsequent denial of care.^74^

C4. In the presence of concurrent neuromusculoskeletal issues, consider a therapeutic trial of chiropractic care, using treatment that has been widely and safely used^8,16,51,75,76^ even when high-level research evidence is currently unavailable.

C4a. Provide parents with the information they need for fully informed consent, including an explanation of supportive measures and collaboration with other health care providers to improve overall health and wellbeing.

#### Infant (children <1 year of age) assessment and treatment—in addition to practices common to all children

IA1. Assess or refer for assessment of the infant's ability to feed by breast or bottle by mother's report. It may include musculoskeletal assessment of cranial symmetry, temporomandibular and/or cervical joint and soft tissue function, as well as any distal site that could refer pain and may affect the infant's ability to feed.^77^

I A1a. Measure and record any cranial asymmetry as treatment plan progresses.^77,78^
I A1b. Observe feeding or the infant's suckling to assess the integrity of oral motor function and make appropriate referrals as necessary for further intervention or neuromuscular reeducation.^79^

IA2. Provide evidence-based advice and information about nutrition (breastfeeding or breast milk substitute).^80^

IA3. Support and encourage parent/infant bonding while providing assessment and treatment because early bonding and attachment are important for development and long-term health.^79,81–83^

IA4. Provide evidence-based advice on safe sleeping for infants.^84^

IA5. Communicate with, comanage with and/or refer infants with disabilities to appropriate providers for more extensive treatment and comanagement.^1^

#### Manual procedures

There are special considerations for use of spinal manipulation and other manual procedures with children.^1,8,16,51,75,76^

SO. Modify manipulative and/or mobilization and soft tissue techniques as appropriate for the child's age and developmental stage.^12,16^

- Sa. Patient size: Modify biomechanical force in proportion to the age and developmental stage of the child.
- Sb. Structural development: Modify manual procedures to ensure the safety of the developing skeleton.
- Sc. Flexibility of joints: Take into account the greater flexibility and lesser muscle mass of children, using gentler and lighter manual procedures.

SO. Patient preferences: Adapt manipulation and soft tissue procedures for the individual child's needs and comfort.

#### Comanagement and referral

CM1. Establish communication and, if possible, collaborative relationships with the child's other healthcare providers in order to effectively and safely co-manage pediatric complaints.^1^

CM2. Establish co-management with other health care providers as appropriate, including but not restricted to:^1,25^

- CM2a. The child is not showing clinically significant improvement after an initial trial of chiropractic care.
- CM2b. The parents of the child request such a co-management approach.
- CM2c. There are significant co-morbidities that are outside the scope of chiropractic practice.
- CM2d. When ordering diagnostic imaging or laboratory studies, forward copies of the results to the child's primary provider for coordination of care, if requested/authorized by the parent/caregiver.
- CM2e. Consider co-management of non-musculoskeletal conditions with the child's primary care provider and/or other providers.
- CM2f. Immediately refer to the appropriate medical specialist when the case history or examination reveal any “red flags” suggestive of serious pathology. A list of these red flags is shown in Figure 1, end of document.

#### Health promotion and disease prevention

##### Primary prevention

PP1. Well child visits are an established aspect of pediatric health care and may be indicated for the purpose of health promotion and clinical assessment of asymptomatic pediatric patients.^22^

PP2. Emphasize disease prevention and health promotion through counseling on physical activity, nutrition, injury prevention, and a generally healthy lifestyle.^20^ These health promotion principles may be addressed through the course of care, and include but are not limited to:

- Adequate age-appropriate physical activity
- Adequate sleep
- Decreased screen time, such as television, mobile/cell phones, electronic games, and computer use
- Healthy diet
- Healthy social relationships
- Injury prevention
- Substance use or abuse prevention (such as caffeinated beverages, alcohol, tobacco,^44^ vaping, marijuana, steroids, and other prescription or illicit drugs). Provide and discuss age-appropriate, readily accessible cessation materials.

PP3. Screen children ages 2–18 years for obesity and offer them lifestyle and dietary advice or consider referring them to a qualified provider for appropriate interventions.^46,85^

PP4. If parents ask about sun exposure for their children, provide them with information from authoritative sources on minimizing exposure to ultraviolet (UV) radiation while maintaining safe exposure for the desirable benefits related to Vitamin D.^45,86,87^

PP5. If parents ask for advice or information about childhood vaccinations, explain that they have the right to make their own health decisions. They should be adequately informed about the benefits and risks to both their child and the broader community associated with these decisions. Consider referral to a health professional whose scope of practice includes vaccinations to address patient questions or concerns.^20^ (See Chiropractic Board of Australia position statement).

##### Secondary prevention—screening

SP1. If parents ask, or if relevant to presenting complaint, provide them with access to resources on correct use of seat belts, car seats, and infant seats, such as those provided by local and national public health agencies.^88,89^

SP2. Any tests or procedures used for public screenings should be based on recognized evidence of their benefit for disease prevention and health promotion.^28^

### Adolescent idiopathic scoliosis

AI1. Screen children for scoliosis; idiopathic scoliosis is more commonly discovered during a child's growth spurt (10 to 15 years old) using established tests, including forward bend test, Scoliometer, Humpometer, plumb line test, or Moiré topography.^41,43,90^

AI2. Refer patients with positive results of scoliosis screening for appropriate imaging examination.^52,65^

AI3. Although there is currently no evidence for effectiveness of spinal manipulation (SMT) on progression or improvement in curvature, evidence is insufficient but favorable for its effectiveness for pain for some adolescents (ages 13–17) with scoliosis. Therefore, consider the risks and benefits of a therapeutic trial of SMT.^50^

### Recognition of family and domestic violence, including child abuse and neglect

FV1. Be alert for signs of family and domestic violence (FDV), including possible child abuse or neglect. These include but are not limited to physical injury, chronic pain, depression, and posttraumatic stress disorder.^47,56,58,91,92^

FV2. Pay particular attention to injuries in various stages of healing and explanations for the injuries being either lacking or incongruous.^56,58,93^

FV3. Where FDV is suspected affecting the welfare of a child, it is necessary to notify the appropriate services.^56,58,93^

FV4. If warranted, refer the family to an appropriately trained mental health practitioner and/or provide access to a resource kit with a list of information and support services.^56,58,93^

## Discussion

The media and fringe medical advocates have sometimes presented the impression that chiropractic care of children, especially infants, has serious safety concerns and an absence of effectiveness.^94^ Indeed, the impetus for this Delphi project was recommendation 6 (p7) of a government inquiry conducted in the Australian state of Victoria in 2019 as a result of media interest.

Consequently, an international, interdisciplinary team, including many with prior experience in formulating best practice guidelines was convened with logistical and financial support from several peak bodies representing the chiropractic profession. This comprehensive Delphi process spanned over 2 years.

The 2009 and updated 2016 recommendations constituted a logical launching point for the project. Due to the robust nature of the original documents, there was little substantive change in many statements with the exception of the sections on health promotion, secondary prevention, and recognition of FDV. Since the SCV inquiry was precipitated around concerns of safety of SMT with children under 12, this review included a deep search for reports of AEs associated with manual care of pediatric patients. Consistent with the SCV inquiry, this study confirmed the rarity of published examples of AEs associated with SMT and manual care.

This Delphi consensus process followed standard methodology by forming an interdisciplinary SC, which included consumers consistent with best practice protocols to evaluate the contemporary literature. The volume of evidence available to clinicians presents a significant challenge since it is not feasible for busy clinicians to review primary research literature routinely.^95^ Thus, CPGs and best practice documents communicate “preprocessed” evidence-based recommendations to clinicians. Where insufficient evidence is available to give graded recommendations, guidelines may offer “consensus-based” recommendations.^96,97^

The guidance statements in this document can primarily form the basis of written information for parents, advising them of proposed benefits and potential risks of intended care. Regulators, practicing chiropractors who treat children, other health care providers, educators, third-party payers, and the general public will also find the content informative and instructive.

These best practices serve also as a scaffold for practitioners who wish to acquire additional knowledge to appropriately care for pediatric subgroups by progressing to postgraduate advanced training or continuing education coursework.

Other health care providers who care for children in collaboration with chiropractors will benefit through enhanced understanding of the nature, scope, and expectations of the pediatric chiropractic encounter. This can lay a foundation for mutual referral and cooperation in the best interest of the children who consult chiropractors.

Health insurers and third-party payers increasingly seek evidence to inform decisions about their medical policies and benefits. Often lacking in this process are the resources and skills needed to synthesize the scientific literature and to gather input from the providers and consumers most affected by their policy decisions. This document represents such a resource.

### Strengths and limitations

A strength of this international project was the involvement of consumers from the initial phase of design through to publication. Approaches that give consumers specific roles or engage them in a formal structure such as a SC^98^ or that enable consumers to set the agenda, develop a shared mission and purpose statements and participate in most stages of the planning, administration, and evaluation make consumer participants feel comfortable with the team and process, maintain consumer involvement throughout the process, and improve the quality of outcomes.^98–104^

We had unprecedented input from consumers through the SC, and we were also able to draw on the consumer submissions that formed an important part of the SCV report. The SC had interdisciplinary expertise from medicine, medical pediatrics, nursing, lactation consultancy, massage, social work, psychology, family therapy, mental health, academics, researchers, and clinicians on both the SC and Delphi panels.

### Limitations

Due to the continuing substantial gaps in the evidence for the effectiveness of SMT care of children, it was important to develop a set of evidence-informed recommendations that are the result of expert opinion achieved through a rigorous consensus process. However, the gaps in the evidence base still represent a limitation to these recommendations because expert consensus is a lower form of evidence to be relied on principally when higher levels of evidence are lacking. It is possible that our limited literature search may have missed relevant citations; however, it is noteworthy that most clinical interventions lack high-quality supportive evidence.^105^

Our recommendations primarily deal with the “typical” patient care journey through history, examination, and management and are not designed to exhaustively cover all possible services chiropractic practitioners may provide for children. We also provided only limited recommendations related to specific childhood age groups; although we discussed infants as a subsection, it was beyond our scope to provide specific guidance for other pediatric age groups (such as toddlers, elementary school, or adolescents).

Another limitation of a study based on consensus is that it is possible that the panelists do not fully represent the general population of chiropractic experts. We did not conduct searches on effectiveness for individual childhood conditions as we adopted the position that delivery of care should be defendable rather than decisive.^13^ Lastly, we did not seek formal input from organizational stakeholders representing educators, third-party payers, legislative bodies, or nonmanual care pediatric organizations. We did not provide any specific recommendations about age-appropriate treatment dosage, frequency, and duration, which were beyond this project's scope.

## Conclusion

This best practice document represents a general framework for an evidence-informed and reasonable approach to safe, defendable management of the general pediatric population by chiropractors. We provide concise statements to guide practitioners along the care journey through communication, consent, health history, examination, screening for red flags, management considerations, and modifications to manual therapies.

## Footnotes

^*^
**Correction added** on November 7, 2023 after first online publication of October 30, 2023: Numerals have been added to the Clinical History section for clarification.

^*^
**Correction added** on November 7, 2023 after first online publication of October 30, 2023: Numerals have been added to the Clinical History section for clarification.
